# Enhancing Transradial Transarterial Microembolization Efficiency and Patient Satisfaction with Oral Benzodiazepine Premedication

**DOI:** 10.3390/diagnostics15131667

**Published:** 2025-06-30

**Authors:** Hsuan-Yin Lin, Ming-Chuan Chiang

**Affiliations:** Department of Medical Imaging, Taichung Veterans General Hospital, 1650 Taiwan Boulevard Sect. 4, Taichung 407219, Taiwan

**Keywords:** transarterial microembolization, transcatheter arterial microembolization, benzodiazepines, chronic pain, vasospasm

## Abstract

**Objectives**: To evaluate the impact of benzodiazepines (BZDs) on pre-procedural anxiety, procedural parameters, post-procedural pain, and satisfaction in transarterial microembolization (TAME). **Methods**: Retrospective analysis of prospectively collected data from 31 patients with refractory upper extremity pain treated with transradial TAME in 2023. Patients were divided into a non-BZD group (*n* = 15; 8 female; age 59.3 ± 9.5 y) and a BZD group (*n* = 16; 10 female; age 53.3 ± 9.9 y). Pre-procedural anxiety (five-point Likert scale), satisfaction (Likert), and pain (Visual Analog Scale, VAS) were assessed. Satisfaction and pain were evaluated immediately and 1 and 3 months post-procedure. **Results**: Baseline demographics, intra-procedural variables, and post-procedural reductions in VAS scores showed no significant intergroup differences (all *p* > 0.05). However, compared to the non-BZD group, the BZD group exhibited a significant reduction in anxiety scores (1.12 vs. 3.80; *p* = 0.04) and procedure time per artery (23.58 vs. 34.81 min; *p* = 0.001). The BZD group also reported significantly higher immediate, short-term, and mid-term satisfaction (4.25 vs. 3.13, *p* = 0.045; 4.69 vs. 3.67, *p* = 0.01; 4.81 vs. 3.80, *p* < 0.001), and a significantly greater proportion exhibited artery diameters ≥ 2 mm (*p* = 0.02). **Conclusions**: TAME with oral BZD premedication significantly improves patient satisfaction, reduces anxiety, and promotes a high proportion of arterial diameters ≥ 2 mm, thereby reducing procedural time.

## 1. Introduction

Transarterial microembolization (TAME) has emerged as a novel treatment option for chronic painful musculoskeletal diseases, which constitute a significant global health burden; although reported prevalence data differ [1,2,3], it is widely acknowledged that approximately one-third of individuals globally experience a chronic, painful musculoskeletal condition [4,5], contributing substantially to global disability and affecting an estimated 1.71 billion people worldwide [6,7]. In recent years, it has gained worldwide recognition for its therapeutic efficacy, particularly for chronic, refractory conditions such as osteoarthritis, adhesive capsulitis, plantar fasciitis, and various inflammatory (‘-itis’) pathologies that do not involve major structural instability [8,9,10,11,12,13,14,15,16,17]. Despite this, transradial puncture, the first step in TAME for upper extremities, presents a significant challenge for many new operators, with published complication rates ranging from 1.8 to 8.0% [18,19,20,21]. Co-occurring depression and anxiety are common in individuals with chronic, intractable pain [22,23]; furthermore, transarterial procedures may exacerbate patient anxiety and induce sympathetic hyperactivity. Elevated sympathetic nerve activity-related vascular constriction can cause conditions such as vasospastic angina, cerebral hypoperfusion, and intra-procedural vasospasm [24,25,26], suggesting that controlling sympathetic tone might optimize intervention outcomes. Benzodiazepines (BZDs) function as GABA agonists, contributing to reduced sympathetic neuronal excitability [27]. Therefore, we hypothesize that premedication with BZDs may mitigate several vasospasm-related challenges during procedures and ultimately improve procedural outcomes.

This study aims to assess the impact of oral BZD premedication on intra-procedural vascular diameter, relevant physiological parameters, and patient satisfaction following TAME procedures.

## 2. Materials and Methods

### 2.1. Patients

The institutional review board approved this retrospective study and waived the requirement for informed consent. This study retrospectively analyzed prospectively collected data from patients with chronic musculoskeletal pain in the upper extremity, recruited from the Radiology Outpatient Department at Taichung Veterans General Hospital between February 2023 and December 2023. Inclusion criteria consisted of age 20 years or older, a diagnosis of chronic musculoskeletal disease affecting the upper extremity, refractoriness to conservative treatment, and predicted responsiveness to TAME based on the reported literature [8,9,11,12,13,14,15,16,17,28]. Exclusion criteria included rheumatoid factor levels of 30 IU/mL or greater, impaired renal function, pregnancy, local infection or malignancy, and a follow-up period of less than 3 months. Thirty-one patients (eighteen female and thirteen male subjects; mean age, 55.9 ± 10.8 years; range 31–71 years) were enrolled in this study. Baseline patient demographic and clinical data are presented in Table 1.

### 2.2. BZD Premedication

Patients in the BZD group were instructed to take their usual prescribed ‘as needed’ (PRN) BZD medication approximately 30 min prior to the procedure. Dosing varied, based on their usage patterns: chronic users, defined as those requiring daily BZD (alprazolam or clonazepam) use, were instructed to take one pill initially, with the option for a second pill fifteen minutes before the procedure if anxiety persisted. Conversely, occasional users, who did not require daily BZD, were advised that a single pill 30 min prior to the procedure would generally suffice. These medications were part of their existing treatment regimen for managing anxiety, insomnia, or depression associated with their chronic pain.

### 2.3. Transarterial Microembolization Procedure

All procedures were performed by the same interventional radiologist (H.Y. Lin), who had four years of experience in embolization procedures and had previously performed 16 transradial upper limb embolization treatments. TAME procedures were performed under local anesthesia, with ultrasound-guided percutaneous access to the ipsilateral radial artery. A 5 Fr. introducer sheath (Prelude^®^, Merit Medical Systems Inc., South Jordan, UT, USA) was used for adhesive capsulitis and lateral/medial epicondylitis, whereas a 20-gauge Jelco needle with a simplified sheathless method was used for hand osteoarthritis treatment. A mixture of 3 mg of Isosorbide dinitrate and 2000 units of heparin was administered intra-arterially via the sheath immediately after it was placed in the radial artery. A Judkins Right 4 Fr. (Terumo Co., Ltd., Tokyo, Japan), RIM Torcon NB^®^ 5 Fr. (COOK Inc., Bloomington, IN, USA), or Sos Omni^®^ 5 Fr. (Angiodynamics Inc., New York, NY, USA) angiocatheter—the choice guided by typical vessel branching patterns at the target site—was introduced into the artery proximal to the target painful joint for digital subtraction angiography (DSA). A 1.9 Fr. Progreat lambda^®^ microcatheter (Terumo Co., Ltd., Tokyo, Japan) was then advanced coaxially through the 4 or 5 Fr. angiocatheter, which had been positioned at the orifice of the target artery, to achieve superselective angiography. Manual contrast injection was performed to visualize the neovascularity and minimize non-target embolization. Embolization was performed with a mixture of 500 mg imipenem/cilastatin sodium (IPM/CS, Imperial Phoenix Co., Ltd., Yangon, Myanmar) and 10 mL of contrast agent. Incremental injections of the mixture, limited to approximately 0.2–0.5 mL, were used to avoid overt embolization until obliteration of the abnormal stain was achieved [29]. The upper limit of the embolic mixture was confined to 2 mL in each terminal arterial branch.

### 2.4. Intra-Procedural Measurements and Outcomes

Technical success was defined as selective catheterization of at least 80% of the typical supplying arteries around the painful joint, followed by embolization of all vessels exhibiting characteristic “evoked pain” or a Moyamoya-like abnormal vascular blush during contrast injection [29]. The diameter of the target artery was routinely determined via pre-procedural ultrasound. Beyond the absolute diameter, a target arterial diameter greater than 2 mm was also evaluated as a key surrogate outcome. This threshold holds clinical significance, as a diameter exceeding 2 mm is required for the 5 Fr. (1.67 mm) sheath used in this study to maintain a sheath-to-artery ratio of less than 1, which was recommended by prior studies for minimizing the risk of post-procedural radial artery occlusion [30,31,32]. The mean heart rate, mean systolic blood pressure (SBP), and procedure time per artery were monitored throughout the procedure. Pre-procedural anxiety and post-procedural satisfaction were measured using a standardized five-point Likert scale [33,34]. This scale, ranging from 1 to 5, separately assessed anxiety (1 = not at all anxious to 5 = extremely anxious) and satisfaction (1 = very dissatisfied to 5 = very satisfied). Participants were instructed to mark the number on the scale that best reflected their current anxiety or satisfaction. Examples such as a score of 1 indicating no anxiety/dissatisfaction, 5 indicating high anxiety/satisfaction, and 3 indicating moderate anxiety/neutral satisfaction were given. Post-procedural pain reduction was assessed using the Visual Analog Scale (VAS) score (mm).

Anxiety was assessed immediately before the procedure. Both patient satisfaction and VAS scores were evaluated at three post-procedural time points: immediately, one month (short-term), and three months (mid-term) after the embolization. The patient satisfaction measure was designed to assess overall comfort and contentment throughout the treatment course, in contrast to measures solely focused on post-procedural pain reduction, a topic that has been investigated and addressed in previous research [8,9,11,12,13,14,15,16,17].

### 2.5. Statistical Analysis

Continuous variables were presented as mean ± standard deviation (SD). Categorical variables were reported as number and percentage. Differences between groups were assessed using the Mann–Whitney U test for continuous variables and the Chi-square test (or Fisher’s exact test, where appropriate) for categorical variables. A two-sided *p*-value of less than 0.05 was considered statistically significant. All analyses were performed using R version 3.1.0, a language and environment for statistical computing (R Foundation for Statistical Computing, Vienna, Austria, https://www.R-project.org/).

## 3. Results

### 3.1. Patients and Procedure

**Figure 1:**
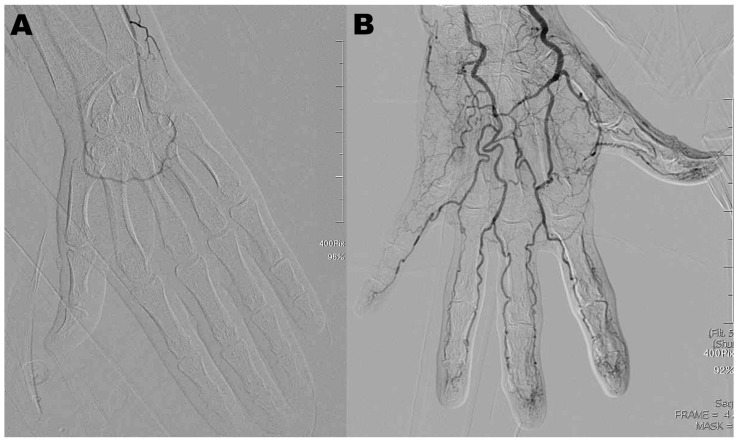
Digital subtraction angiography in a 31-year-old man ((**A**), non-BZD group) and a 61-year-old woman ((**B**), BZD group) with hand osteoarthritis. Severe intra-procedural distal vascular spasm, unresponsive to multiple doses of intra-arterial vasodilator injection, was observed in the patient without BZD premedication.

Table 1 summarizes the baseline demographic and clinical data of patients in the BZD and non-BZD groups. Thirty-one patients with various diagnoses (hand OA, *n* = 15; adhesive capsulitis, *n* = 7; lateral epicondylitis, *n* = 3; medial and lateral epicondylitis, *n* = 3, psoriatic arthritis, *n* = 2, medial epicondylitis, *n* = 1) meeting inclusion criteria and exhibiting no exclusion criteria, were treated with TAME. Technical success was achieved in 96.8% of the cases. The only technical failure among the 31 patients occurred in the non-BZD group and was attributed to significant intra-procedural vasospasm that persisted despite repeated intra-arterial vasodilator administration (Figure 1). Most intra-procedural vasospasm was successfully managed with intra-arterial isosorbide dinitrate infusion. However, some patients subsequently developed dizziness, headache, or nausea.

### 3.2. Baseline and Intra-Procedural Outcomes

**Figure 2:**
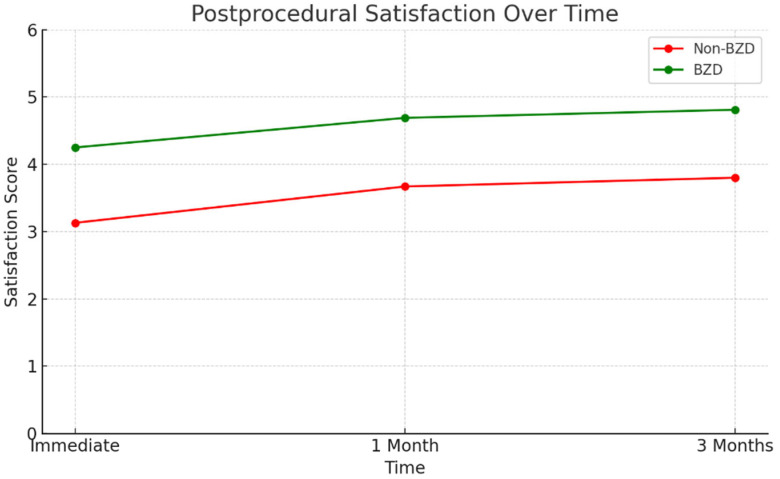
Post-procedural patient satisfaction over time in BZD and Non-BZD groups.

As presented in Table 2 and Table 3 and Figure 2, statistical analysis revealed no significant differences between the groups in terms of baseline demographics, specifically age (58.9 ± 11.6 vs. 61.6 ± 8.9, *p* = 0.11) and sex (Female 62.5% vs. 53.3%, *p* = 0.78). Similarly, there were no significant differences in whether the procedure was performed on the dominant hand (50% vs. 40%, *p* = 0.58) or in intra-procedural physiological parameters, including mean heart rate (71.0 ± 10.5 vs. 78.6 ± 12.2, *p* = 0.37) and mean SBP (130.2 ± 15.8 vs. 159.0 ± 18.5, *p* = 0.44).

**Figure 3:**
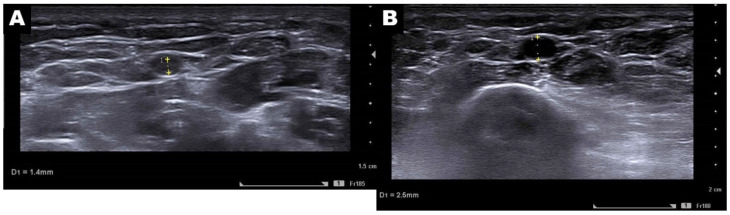
Pre-procedural ultrasonography demonstrating the target puncture artery in a 52-year-old woman ((**A**), non-BZD group) with hand osteoarthritis and a 68-year-old woman ((**B**), BZD group) with adhesive capsulitis. The diameter of the target puncture artery measured 1.4 mm in the non-BZD patient and 2.5 mm in the patient after BZD premedication. The latter diameter (2.5 mm) is optimal for procedures using a 5 Fr. (1.67 mm) sheath, as a diameter > 2 mm is required to maintain a sheath-to-artery ratio of less than 1 to minimize the risk of post-procedural radial artery occlusion.

There was no statistically significant difference in post-procedural VAS score reduction between the BZD and non-BZD groups at either short-term or mid-term follow-up. In contrast, compared to the non-BZD group, patients in the BZD group exhibited a statistically significant reduction in anxiety scores (1.12 ± 0.75 vs. 3.80 ± 1.20, *p* = 0.04) and a significant decrease in the mean procedure time per artery (23.58 ± 6.48 min vs. 34.81 ± 7.92 min, *p* = 0.001). The BZD group also reported significantly higher immediate (4.25 ± 0.80 vs. 3.13 ± 0.90, *p* = 0.045), short-term (4.69 ± 0.85 vs. 3.67 ± 1.00, *p* = 0.01), and mid-term (4.81 ± 0.70 vs. 3.80 ± 1.10, *p* < 0.001) satisfaction scores. Finally, as illustrated in Figure 3 and Table 3, the BZD group exhibited a significantly greater proportion of patients with a pre-procedural puncture artery diameter of 2 mm or greater (*p* = 0.02).

The line graph compares patient satisfaction scores (measured by standardized five-point Likert scale) between the BZD (green line) and Non-BZD (red line) groups at three time points: immediate, one month, and three months post-procedure.

### 3.3. Adverse Events

There were no procedure-related major adverse events. Notably, no adverse episodes attributable to BZD premedication, such as falls or prolonged/unresponsive somnolence, occurred.

During the follow-up period, there were no reports of new pain, muscle weakness, or persistent skin color changes in any embolized territory. However, one patient experienced transient numbness and paresthesia in the fingertips following embolization, resolving within one week with oral gabapentin treatment, following strategies described in previous articles [28,35].

## 4. Discussion

The efficacy and safety of TAME for treating a variety of chronic painful musculoskeletal diseases are well-recognized through accumulating evidence [8,9,10,11,12,13,14,15,16,17]. However, despite its recognized therapeutic potential, less attention has been paid to mitigating the learning curve and broadening its adoption [29,36,37]. Studies focused on procedural refinements such as premedication or troubleshooting strategies are therefore valuable for improving TAME accessibility and outcome consistency.

This study provides preliminary evidence suggesting that oral BZD premedication, a notably simple intervention, significantly enhances the patient experience during transradial TAME, primarily through reduced pre-procedural anxiety and improved post-procedural satisfaction at immediate, short-term, and mid-term follow-ups. Furthermore, our findings indicate an association between BZD premedication and procedural advantages, namely a higher proportion of patients exhibiting puncture artery diameters ≥ 2 mm and a consequently shorter mean procedure time per artery.

BZD premedication provided significant benefits to patient comfort and procedural efficiency. Crucially, TAME’s primary effectiveness in reducing pain, evidenced by comparable post-procedural VAS scores, remained undiminished. BZDs thus functioned as a valuable supportive measure, improving the peri-procedural experience and technical facilitation, without compromising the therapeutic effect of TAME.

A notable aspect of this study is that all procedures were performed by a single, experienced interventional radiologist within a relatively short timeframe (10 months). While utilizing a single operator might introduce potential operator-specific bias, it concurrently offers a significant advantage by eliminating inter-operator variability. Differences in technical skill, procedural pacing, and even communication style among multiple operators could otherwise confound the assessment of factors like procedure time, patient anxiety, and satisfaction. Additionally, the condensed data-collection period helps mitigate the potential confounding effects of a learning curve, where an operator’s improving proficiency over a longer time might influence later results. Therefore, the single-operator design, in this context, enhances the internal consistency of the procedural performance and its relation to the premedication strategy.

Intra-procedural vasospasm remains a recognized challenge in transradial access procedures [24,25,26,38]. While the administration of intra-arterial vasodilators, such as nitroglycerin (NTG) or isosorbide dinitrate used in our protocol, is a common strategy to manage vasospasm once it occurs, this reactive approach is not without potential drawbacks [39,40]. Previous reports have documented complications associated with intra-arterial vasodilator infusion, including pain in the affected limb, nausea/vomiting, hemodynamic instability (significant hypotension requiring vasopressor support), and in rare, severe instances, even the need for endotracheal intubation [38,41]. Conversely, proactive BZD premedication reduces patient anxiety, potentially lessening the sympathetic hyperactivity [27], which drives vasoconstriction [23,24,25]. This strategy aims to prevent or reduce the severity of vasospasm, possibly decreasing the need for intra-procedural vasodilators and their associated risks. The higher proportion of artery diameters ≥ 2 mm and shorter procedure time per artery in the BZD group support this hypothesis, suggesting a more favorable vascular environment facilitated by reduced anxiety and sympathetic tone. Therefore, we propose that managing anxiety and its physiological effects is a potentially safer, more fundamental, and effective method for improving vascular conditions during transradial TAME.

There are several limitations in this study. Firstly, the study design is a retrospective analysis of prospectively collected data. Although baseline demographics were largely comparable, the lack of randomization introduces the possibility of selection bias or unmeasured confounding variables influencing outcomes. Secondly, the study was conducted at a single center with a single operator, which, while enhancing internal consistency as discussed, limits the generalizability of the findings to other institutions or operators with different patient populations or skill sets. However, it should be noted that the specific premedication strategy using oral BZDs is straightforward and readily reproducible in most clinical settings. Thirdly, the sample size is relatively small (*n* = 31), which may limit the statistical power to detect more subtle differences or increase the risk of Type II error. Finally, the assessment of anxiety and satisfaction relied on subjective Likert scales, which, although standardized, are inherently subjective and potentially influenced by patient expectations or recall bias.

Despite these limitations, this study is, to our knowledge, the first to specifically assess the impact of BZD premedication on procedural parameters and patient-reported outcomes specifically for transradial TAME. Based on these preliminary findings, oral BZD premedication represents a readily implementable strategy that clinicians performing transradial TAME procedures may consider to enhance patient comfort and potentially facilitate the procedure workflow. Further investigation, preferably using a prospective, randomized, multicenter design with a larger sample size, and incorporating careful screening for potential BZD dependence, would be valuable to confirm these preliminary results and further explore optimal BZD dosing strategies.

## 5. Conclusions

Oral BZD premedication in transradial TAME procedures is associated with statistically significant improvements in patient satisfaction and a reduction in anxiety. This readily implemented premedication strategy is further associated with a higher proportion of patients exhibiting artery diameters ≥ 2 mm, potentially facilitating the procedure and reducing procedural time.

## Figures and Tables

**Table 1:** Baseline patient demographic and clinical data.

Non-BZD Group					BZD Group				
Pt	Sex	Age (yr)	Diagnosis	Supplying Arteries (*n*)	Pt	Sex	Age (yr)	Diagnosis	Supplying Arteries (*n*)
1	F	54	Hand osteoarthritis (OA)	2	1	M	40	Medial and lateral epicondylitis	7
2	F	56	Hand OA	2	2	F	52	Medial epicondylitis	3
3	M	54	Adhesive capsulitis	6	3	M	39	Medial and lateral epicondylitis	7
4	M	60	Lateral epicondylitis	4	4	F	53	Hand OA	2
5	F	61	Adhesive capsulitis	6	5	F	62	Hand OA	2
6	M	71	Adhesive capsulitis	6	6	F	61	Hand OA	2
7	F	67	Hand OA	2	7	F	66	Hand OA	2
8	F	65	Hand OA	2	8	M	58	Adhesive capsulitis	6
9	F	64	Adhesive capsulitis	6	9	F	68	Adhesive capsulitis	6
10	M	59	Adhesive capsulitis	6	10	M	43	Medial and lateral epicondylitis	7
11	F	64	Psoriatic arthritis	2	11	M	43	Lateral epicondylitis	4
12	F	61	Psoriatic arthritis	2	12	F	57	Hand OA	2
13	M	57	Hand OA	2	13	F	59	Hand OA	2
14	M	60	Hand OA	2	14	F	67	Hand OA	2
15	M	31	Hand OA	2	15	F	61	Hand OA	2
					16	M	43	Lateral epicondylitis	4

**Table 2:** Comparison of baseline patient characteristics and procedural laterality.

Variables	Non-BZD (*n* = 15)	BZD (*n* = 16)	*p* Value
Age (yr +/− SD)	61.6 ± 8.9	58.9 ± 11.6	0.11
Sex (Female, *n*, %)	8 (53.3)	10 (62.5)	0.78
Dominant hand (*n*, %)	6 (40.0)	8 (50.0)	0.58

**Table 3:** Intra-procedural measurements and post-procedural patient satisfaction.

Variables	Non-BZD (*n* = 15)	BZD (*n* = 16)	*p* Value
**Intra-procedural measurements**			
Patient anxiety	3.80 ± 1.20	1.12 ± 0.75	0.04
Arterial diameter ≥ 2 mm (*n*, %)	6 (40.0)	14 (87.5)	0.02
Mean heart rate (bpm)	78.6 ± 12.2	71.0 ± 10.5	0.37
Mean systolic blood pressure (mmHg)	159.0 ± 18.5	130.2 ± 15.8	0.44
Procedure time/per artery (mins)	34.81 ± 7.92	23.58 ± 6.48	0.001
**Post-procedural Satisfaction**			
Immediate	3.13 ± 0.90	4.25 ± 0.80	0.045
Short-term (one month)	3.67 ± 1.00	4.69 ± 0.85	0.01
Mid-term (three months)	3.80 ± 1.10	4.81 ± 0.70	<0.001
**Post-procedural reduction in VAS ^†^ score (mm)**			
Short-term (one month)	4.6 ± 2.77	5.3 ± 1.74	0.32
Mid-term (three months)	6.2 ± 2.24	7.4 ± 1.26	0.10

^†^ VAS: Visual Analog Scale.

## Data Availability

The original contributions presented in this study are included in the article. Further inquiries can be directed to the corresponding authors.
